# Socio-economic status is inversely related to bed net use in Gabon

**DOI:** 10.1186/1475-2875-7-60

**Published:** 2008-04-18

**Authors:** Julia N Goesch, Norbert G Schwarz, Marie-Luise Decker, Sunny Oyakhirome, Lea B Borchert, Ulrich D Kombila, Marc Poetschke, Bertrand Lell, Saadou Issifou, Peter G Kremsner, Martin P Grobusch

**Affiliations:** 1 Medical Research Unit, Albert Schweitzer Hospital, Lambaréné, Gabon; 2 Department of Parasitology, Institute of Tropical Medicine, University of Tübingen, Germany; 3 Infectious Diseases Unit, Division of Clinical Microbiology and Infectious Diseases, National Health Laboratory Service and School of Pathology, Faculty of Health Sciences, University of the Witwatersrand, Johannesburg, South Africa

## Abstract

**Background:**

Insecticide-treated bed nets (ITNs) range among the most effective measures of malaria prophylaxis, yet their implementation level in sub-Saharan Africa is still low. The goal of this study was to investigate the influence of socio-economic factors on the use of bed nets by mothers in Gabon.

**Methods:**

A cross-sectional study was conducted completing pre-tested, interviewer-administered questionnaires exploring socioeconomic proxy measures with 397 mothers or guardians of young children. Respondents were grouped according to their socio-economic situation, using scores. The condition of the bed nets was evaluated during a home visit.

**Results:**

Socio-economic factors of wellbeing were negatively associated with bed net use, such as living in a stone house (OR 0.26, 95% CI 0.14–0.48), running water in the house (OR 0.44, 95% CI 0.21–0.92), shower/flush toilet in the house (OR 0.39/0.34, 95% CI 0.21–0.75/0.16–0.73), ownership of a freezer (OR 0.50, 95% CI 0.26–0.96) and belonging to the highest group in the economic score (OR 0.32, 95% CI 0.15–0.67). In contrast, similar factors were positively associated with a good maintenance condition of the bed nets: higher monthly income (OR 5.64, 95% CI 2.41–13.19) and belonging to the highest group in the economic score (OR 2.55, 95% CI 1.19 – 5.45).

**Conclusion:**

Among the poorest families in Lambaréné the coverage with untreated nets (UTNs) is the highest, but the condition of these UTNs is the worst. To achieve a broad implementation of ITNs in Lambaréné, there is an urgent need for educational programmes as well as need-tailored marketing strategies for ITNs.

## Background

*Plasmodium falciparum *malaria causes between 350 and 500 million clinical episodes and over one million deaths annually [1]. Children and pregnant women are the most vulnerable group and most endangered by the disease [2]. Insecticide-treated bed nets (ITNs) have proven to be effective [3] and also cost-effective [4,5] preventive measures against *P. falciparum *infection, yet their implementation in sub-Saharan Africa is still low [6-9]. New options, which could supersede the necessity of regular re-impregnation in the future, are the development of long-lasting impregnated bed nets (LLINS) [10-12] and long-lasting impregnation tablets, which provide insecticidal activity even after 30 washing cycles [13]. In order to approach the goal of a broad implementation of ITNs, it is essential to identify the factors in favour of their use among the population of endemic areas. Socio-economic factors have been investigated in several studies, focusing on their relation to the severity of disease [14-16], the possession of bed nets [17,18] or the willingness to pay for bed nets [19]. The goal of this study was to investigate the influence of socio-economic factors on malaria prophylaxis – especially the actual use of bed nets by mothers of young children in Gabon.

## Methods

### Study site

This study was conducted at the Medical Research Unit of the Albert Schweitzer Hospital in Lambaréné, Gabon. The interviews for the study took place between December 2004 and July 2005. Lambaréné is a small town of approximately 25,000 inhabitants, located about 250 km from the coastline and 80 km from the equator. The climate is tropical, Lambaréné is surrounded by central African rain forest, malaria transmission is perennial, with little seasonal variation. The entomological inoculation rate is about 50 infective bites per person per year, most infections being caused by *P. falciparum *[20,21].

### Study population

The study subjects were children of three to 24 months of age, who were enrolled in a prospective Intermittent Preventive Treatment for infants (IPTi) trial [22]. The inclusion and exclusion criteria for the children correspond to those of the IPTi trial, 397 mothers or guardians were selected randomly for the interviews, informed consent was obtained from each of them beforehand. The Ethics Committee of the International Foundation of the Albert Schweitzer Hospital in Lambaréné approved of the questionnaire and the study procedures.

### Questionnaire

The questionnaire included 60 questions, 40 of which were related to malaria prophylaxis or indicators of socio-economic status. Respondents were interviewed by especially trained interviewers, using default wording. The questionnaire consisted mainly of closed questions. Respondents were not prompted, only spontaneously given answers were marked on the questionnaire sheet by the interviewer. Data entry was limited to two persons. The interviews took place at the home of the respondent during a clinical visit of the child enrolled in the ITPi study, or at the medical ward when mother and child were visiting for a scheduled routine control. All subjects of the IPTi study had the same number of appointments and, therefore, the same chance of being included in the sub-study.

### Evaluation of bed nets

To measure the proportion of children not sleeping under a bed net, it was asked whether the child had slept under a bed net the night before the interview. This was the main outcome measure of the study. Condition and installation of bed nets were evaluated during a home visit in order to compare not only the percentages of bed nets used, but also the percentages of effectively used bed nets. The bed net chosen for evaluation was always the one under which the study child had slept the previous night. Bed nets were labeled *sufficient*, if they did not have holes (regardless of their impregnation status) or *insufficient*, if any holes were observed by the investigator.

### Economic score

Substitute measures for the social and the economic situation of a family were collected during the interview. Two simple scores, one for the social and one for the economic situation, were developed.

The economic score was calculated using the following variables, which are commonly used as markers for the economic wellbeing of a family:

Living in a stone house

Electricity in the house

Possession of a television set

Possession of a freezer

Running water in the house

Shower in the house or next to it

Flush toilet in the house

Monthly income self-estimated above 100,000 CFA (~150 Euros)

One point was granted for each positive answer, the respondents were stratified accordingly into three equally sized groups using percentiles, the highest group being the one with most positive answers.

### Social score

The following outcomes were considered positive and granted one point in the social score:

Mothers age: respondent aged 18 or above

School education: 6 years or more

Respondent being a skilled worker

Respondent being married

Partner of the respondent being the biological father of the study child

Father of the study child living in the same house with the family

Fathers age: father of the study child aged 25 or above

Number of children in the household not above 4

Likewise, the respondents were stratified into three equally sized groups, the highest group being the one with most positive answers.

### Data analysis

Statistical analysis was performed using Stata9^® ^(Stata corp., College Station, Texas, USA) and SPSS11.0^® ^(Mac OS X Version, Software MacKiev) statistical software. To verify the association between categorical variables, the chi square test was employed. If a cell had an expected frequency below 5, Fisher's exact test was used. For each of the variables we calculated odds ratios using univariate logistic regression and adjusted odds ratios from a full model containing all exposure variables with a p-value below 0.1 in the univariate analysis. There was no further backwards modeling, as the variables are only proxy measures for the economic situation. Reducing the number of variables in a backwards modeling approach may give the potentially wrong impression of a causal relationship between the few variables remaining in the model and the outcome. The economic score and the social score were excluded from the multivariate model.

## Results

### Socio-economic factors

All 397 respondents were questioned about social and economical aspects of their daily life. 15% of the respondents were still students. 4% did not receive any formal education at all. Most of the others went to school for more than four (84%) but less than six years (78%) and only 1% had graduated from high school. 18% were skilled workers who had learned a profession. Only 25% of the respondents were married (legally, traditionally or at a church), but 58% lived with the father of the study child (usually the youngest child of the respondent). Houses were frequently shared with other family members and their children, forming one household with a mean number of four adults and five children per household.

21% of the participating families lived in stone houses. The majority (61%) inhabited wooden houses with a solid basement made of cement and a tin roof. 18% dwelled in the poorest types of houses, which were wooden huts with a beaten-earth floor, or in one case a traditional loam house. 83% of the houses had access to electricity and 68% respectively 59% owned electrical devices like a television set or a freezer. 14% of the houses were provided with running water, most other families collected their water from a public water pump (83%). Very few used well or river water. 19% respectively 10% stated, that they had a shower or a flush toilet either in the house or next to it. 73% of the respondents estimated the monthly income of their family being less than 100,000 CFA, which at the time of writing equalled approximately 150 euros.

### Implementation of prophylactic means in the study population

The respondents were questioned about their preventive behaviour and the prophylactic means they used to protect themselves and the study child. The knowledge about the transmission of malaria was generally good: 94% of the respondents knew that malaria is transmitted by mosquitoes and 93% spontaneously named bed nets when asked for means of protection. 349 respondents (88%) stated that their child regularly sleeps under a bed net. 213 of these bed nets were evaluated during a home visit. The remaining could not be evaluated due to logistic reasons, because the family moved or the study child dropped out of the umbrella study. Usually one bed net was shared between two or three people, for instance a couple and their youngest child sleeping in one bed together. The bed nets encountered during the evaluation campaign were mostly UTNs (untreated nets), only 6.4% had ever been impregnated with insecticides, but 97% of the respondents would have been willing to take part in an impregnation intervention, if provided for free. 70% of the bed nets were in a good condition, having no holes (and were therefore labelled *sufficient*), whereas 10% had small holes, 15% had big holes (more than 1 square cm) and 5% were labelled *absolutely useless *because they were so torn and dotted with holes that they would hardly provide any protection from mosquito bites. The installation was mostly demonstrated correctly by the respondent, closing the eaves tightly and inserting the bottom part of the bed net between the mattress and the bed frame (85%).

Other prophylactic means used in the study population (as stated by the respondents) included insecticidal spray (50%), chemoprophylaxis – usually with chloroquine – (10%) and long clothing after dawn (3%). 17% of all respondents and 37% of the ones who did not use bed nets believed to be protected from malaria infection by the use of ventilators or air condition. In the group of the nonusers this was most frequently stated as the reason for not using bed nets, followed by the aggravation of high night time temperatures when sleeping under a bed net (28%), economic reasons (18%) and the belief to be well protected by insecticidal spray alone (12%). 26% of the nonusers stated to be willing to use a bed net if it was provided for free.

### Association of single socio-economic factors with the use of bed nets

Table 1 shows the distribution of socio-economic variables in relation to the question if the study child slept or did not sleep under a bed net the previous night. The following factors were significantly associated with the use of bed nets: mother being a skilled worker, living in a wood or loam house, having no running water in the house, lack of a shower/flush toilet in the house and not owning a freezer. Respondents who had a monthly income below 100,000 CFA who had no access to electricity and who were 18 years old or older were also more likely to use bed nets but did not reach statistical significance.

**Table 1:** Determinants of bed net use

	n	child sleeping under bed net [%]	odds ratio	95% CI	p-value
Overall	397	88%			
**Mothers age**	388				
</= 18	14.9%	83%	1.0		
>/= 18	85.1%	88%	1.47	0.69–3.13	0.32
**School education**	390				
<6 years	77.6%	88%	1.0		
>6 years	22.4%	86%	0.88	0.44–1.78	0.73
**Skilled worker**	388				
No	82.0%	85%	1.0		
Yes	18.0%	97%	6.04	1.43–25.49	0.003*
**Marital status**	391				
Solitary	75.5%	87%	1.0		
Married	24.6%	88%	1.04	0.52–2.07	0.92
**House**	395				
Made of wood	79.0%	91%	1.0		
Made of stone	21.0%	72%	0.26	0.14–0.48	<0.0005*
**Electricity**	392				
No	17.1%	94%	1.0		
Yes	82.9%	86%	0.38	0.13–1.08	0.07
**Running water**	392				
No	86.2%	89%	1.0		
Yes	13.8%	78%	0.44	0.21–0.92	0.03
**Shower**	392				
No	81.4%	89%	1.0		
Yes	18.6%	77%	0.39	0.21–0.75	0.005
**Flush toilet**	392				
No	89.8%	89%	1.0		
Yes	10.2%	73%	0.34	0.16–0.73	0.006
**TV**	392				
No	31.9%	88%	1.0		
Yes	68.1%	87%	0.88	0.46–1.67	0.68
**Freezer**	392				
No	41.1%	91%	1.0		
Yes	58.9%	84%	0.50	0.26–0.96	0.04
**Monthly income**	397				
<100 000 CFA	72.8%	89%	1.0		
>100 000 CFA	27.2%	82%	0.58	0.31–1.08	0.09
**Social Score**	384				
Lowest group	36.4%	88%	1.0		
Middle group	24.5%	84%	0.72	0.34–1.53	
Highest group	38.8%	89%	1.06	0.52–2.18	0.56
**Economic Score**	392				
Lowest group	36.2%	92%	1.0		
Middle group	26.8%	90%	0.80	0.33–1.95	
Highest group	37.0%	79%	0.32	0.15–0.67	0.003

(* Fisher' exact test)

### Association of single socio-economic factors with the condition of bed nets

Table 2 shows the distribution of socio-economic factors regarding the condition of the bed nets. A sufficient condition of the bed net was significantly associated with a monthly income above 100,000 CFA.

**Table 2:** Determinants for index net being in a sufficient condition

	n	bed nets labelled *sufficient *[%]	odds ratio	95% CI	p-value
Overall	213	70%			
**Mothers age**	207				
</= 18	16.4%	71%	1.0		
>/= 18	83.6%	70%	0.97	0.43–2.17	0.94
**School education**	211				
<6 years	77.7%	71%	1.0		
>6 years	22.3%	70%	0.98	0.48–1.98	0.95
**Skilled worker**	207				
No	78.7%	71%	1.0		
Yes	21.3%	71%	1.01	0.49–2.09	0.99
**Marital status**	210				
Solitary	24.8%	68%	1.0		
Married	75.2%	75%	1.39	0.68–2.83	0.37
**House**	212				
Made of wood	82.1%	69%	1.0		
Made of stone	17.9%	79%	1.69	0.73–3.92	0.22
**Electricity**	209				
No	18.7%	69%	1.0		
Yes	81.3%	72%	1.13	0.53–2.41	0.75
**Running water**	210				
No	87.6%	69%	1.0		
Yes	12.4%	80%	1.78	0.64–4.98	0.35*
**Shower**	209				
No	82.3%	69%	1.0		
Yes	17.7%	81%	1.91	0.79–4.62	0.15
**Flush toilet**	209				
No	91.4%	70%	1.0		
Yes	8.6%	83%	2.13	0.59–7.63	0.29*
**TV**	209				
No	34.5%	64%	1.0		
Yes	65.5%	75%	1.71	0.92–3.18	0.09
**Freezer**	209				
No	41.2%	70%	1.0		
Yes	58.8%	72%	1.13	0.62–2.08	0.68
**Monthly income**	213				
<100 000 CFA	67.6%	61%	1.0		
>100 000 CFA	32.4%	90%	5.64	2.41–13.19	**<0.0005**
**Social Score**	208				
Lowest group	38.8%	65%	1.0		
Middle group	23.9%	65%	0.99	0.47–2.06	
Highest group	37.3%	81%	2.23	1.08–4.61	0.06
**Economic Score**	209				
Lowest group	36.8%	66%	1.0		
Middle group	25.4%	61%	0.80	0.39–1.65	
Highest group	37.8%	83%	2.55	1.19–5.45	**0.01**

(*Fisher's exact test)

### Association of socio-economic scores with the use and condition of bed nets

When stratified into three groups according to a score composed of several economic factors (economic score), the respondents belonging to the most affluent group used significantly less bed nets (79%) than the middle (90%) and the poorest group (92%). In contrast, the condition of the bed nets as found during the evaluation campaign was significantly better in the most affluent group. Here, 83% of the bed nets were in a sufficient condition while only 61%/66% were labeled *sufficient *in the middle and the poorest group. A high rating in the social score also favoured a good condition of the bed net (even though not statistically significant, p = 0.06) whereas it had no apparent relation to the percentage of bed net users (Tables 1 and 2). There was a trend towards better prophylactic behaviour in the groups with better knowledge about malaria.

### Multivariate analysis

Multivariate logistic regression analysis of factors significant in the univariate analysis was performed. Table 3 shows the corresponding adjusted odds ratios for all exposure variables with a p-value below 0.1 in the univariate analysis. Most variables became non-significant after mutual adjustment. The association of the variable skilled worker to bed net use became more pronounced.

**Table 3:** Determinants of net use in a multivariate analysis

	OR	95% CI	p-value
Overall	376		
**Skilled worker**			
No	1.0		
Yes	6.95	1.59–30.5	0.01
**House**			
Made of wood	1.0		
Made of stone	0.48	0.22–1.07	0.07
**Electricity**			
No	1.0		
Yes	0.65	0.19–2.22	0.49
**Running water**			
No	1.0		
Yes	0.87	0.32–2.35	0.79
**Shower**			
No	1.0		
Yes	0.83	0.32–2.11	0.69
**Flush toilet**			
No	1.0		
Yes	0.71	0.20–2.48	0.59
**Freezer**			
No	1.0		
Yes	0.90	0.40–2.04	0.80
**Monthly income**			
<100,000 CFA		1.0	
>100,000 CFA	0.60	0.30–1.19	0.15

(The table and the adjusted model contain all variables with a p-value smaller than 0.1 in the univariate analysis)

## Discussion

### Malaria and socioeconomic factors

This study confirms the influence of socio-economic factors on prophylactic behaviour, thus being in accordance with previous studies, which identified an association of socio-economic factors with several aspects of the disease and its prophylaxis. Carme *et al *found a significant association of socio-economic factors with the occurrence of cerebral malaria in the Republic of Congo [14]. Koram *et al *described an association of poor quality housing, crowding and travel to rural areas with the incidence of malaria in Gambian children in 1995 [23], but they did not confirm the association of socio-economic factors with the severity of the disease [15]. In 1998, Luckner *et al *stated a tendency towards longer infection-free intervals in Gabonese children who lived in stone houses and whose mothers were older than 24 years and had over six years of formal education [16]. In that study, the influence of socio-economic factors on the outcomes severe malaria and time to first re-infection was not significant. In a Ugandan study from 2001, higher scores in a socio-economic index, composed of several factors, were associated with the possession of bed nets: In that study of Nuwaha *et al *the determinants being most strongly associated with the possession of at least one bed net in the household were a permanent residence and the opinion that bed nets are worth their cost [17]. In a Nigerian study of 2003, the willingness to pay for bed nets was the lowest among lower socio-economic groups [19]. In 2005, Osero *et al *interviewed 400 mothers of a Kenyan population among which bed net use was low (34%) and found that the possession of ITNs was significantly related with the mothers' education, occupation and knowledge [18]. Another Kenyan Study (2006), showed that homestead wealth, travel time to nearest market and mothers education were associated with the use of bed nets by children under five years of age [24].

According to different settings and study protocols, these findings are not always consistent but, regarding the overall pattern, the occurrence of malaria or its consequences was usually associated with a low socio-economic status or its substitute measures, while the possession of bed nets, the willingness to pay for them or their actual use were associated with factors in favour of a higher socio-economic status. In contrast, in the present study which measured the actual use of bed nets in the age group of children between three and 24 months, the use of bed nets was inversely related to the socio-economic status of the mother or caretaker: the percentage of bed net users was significantly higher among families that were living under very simple conditions in a bad economic situation (lowest group of the economic score).

### Insect nuisance hypothesis

One possible explanation for this phenomenon is the pest of insects being much worse in poorer neighbourhoods, which are often located in suburban or rural areas. Additionally, the houses in these areas often have open eaves and lack mosquito netting at the windows, which also leads to a much greater nuisance by insect bites and explains why the inhabitants are much more likely to use bed nets to secure their sleep. In contrast, respondents living under relatively comfortable conditions in houses that allow little entrance for mosquitoes and owning ventilators or air conditioning, often believed to be sufficiently protected from mosquito bites and therefore abandoned their bed nets. This is in accordance with a Ghanaian study which indicated far higher bed net use and ownership in rural than urban areas "which was related partly to perceived affordability and partly to the different contexts of and reasons for avoiding mosquitoes" [25].

### Scores and statistics

The evaluation of several single factors which are commonly used as substitute measures for wealth and a higher socio-economic status corroborates the trend shown in the composed economic score: Among the factors which were most significantly associated with bed net use in the univariate analysis were a poorer house type and the lack of luxury goods such as a shower or a flush toilet. The fact that these variables became non-significant after mutual adjustment shows that none of the variables should be seen as a single causal risk factor, rather than that they are proxy measures for wealth versus poverty. The association of the variable skilled worker to bed net use became more pronounced after adjusting for the other socioeconomic proxy measures. This suggests an effect of an archived job qualification on bed net use that is independent from economic wellbeing.

Factors representing the most disadvantaging living conditions seem to be predictors of bed net use by mothers in Gabon. The Economic score generated for this study has been kept simple. An economic score generated using Principal Component Analysis (PCA) [26,27] including the same economic proxy measures showed 99.3% agreement for the highest group, 100% for the middle group and 99.3% for the lowest group (Data not shown).

### Bed net use and bed net condition

As opposed to the percentage of bed net users, the maintenance condition of the bed nets was straight related to the socio-economic status of the respondents. A higher monthly income and belonging to a higher group in the economic score were significantly associated with a sufficient condition of the bed net under which the study child slept.

The evaluation of the bed nets included two steps: Primarily the number of bed nets in the household, the use of these bed nets by the study child and the impregnation status of the bed net were documented as suggested by the monitoring and evaluation refenrence group (MERG) of the Roll Back Malaria Partnership [28]. These recommendations all focus on ITNs and do not include an evaluation of the condition of the net regarding holes and correct installation. The criteria we used to assign each bed net to the groups *sufficient *or *insufficient *are described in the methods section. Families living under slightly more favourable economic circumstances usually possessed bed nets which were in a good condition, while the poorest families owned most of the badly torn nets labelled as *absolutely useless*.

This is most likely due to the fact that the maintenance of bed nets costs money, and that poor families cannot afford to replace their bed nets regularly when they are worn out and holey. These families would largely benefit from free distribution or social marketing interventions offering ITNs to subsidized prices, such as 1997 in Tanzania, where the ratio of net ownership among the poorest to least poor increased from 0.3 to 0.6 in 2000 after the introduction of a social marketing programme [29] or in Eritrea, where after a large-scale ITN distribution programme in 2002 and 2003 the Abuja target was exceeded with 76% of children under five years sleeping under ITNs [30].

### ITN use among the study population

In the present study, the percentage of ITNs among the bed nets was quite low (6.4% compared to 13% estimated by Webster *et al *[8]), even though 45% of the respondents knew what the word impregnation means. Ninety-seven percent stated that they would be willing to take part in an impregnation project if offered free of charge. UTNs are widely distributed in the private sector in Gabon, while ITNs can only be purchased in the capital Libreville and are not affordable for most parts of the population. LLINs are not available at the local markets yet. This is in accordance with the findings of Webster *et al *[8], who concluded that the contribution of commercial markets for UTNs to the bed net coverage in African countries has been underestimated in comparison to the deliverance of ITNs by public health systems and projects. No social marketing campaigns or free-distribution schemes for ITNs or LLINs have been undertaken in the Lambaréné area so far.

## Conclusion

Among the poorest families in Lambaréné, the coverage with untreated nets (UTNs) is the highest, but the condition of the UTNs is worst. To achieve a broad implementation of impregnated bed nets (ITNs) in Lambaréné, there is an urgent need of social marketing of ITNs or LLINs and free impregnation possibilities for existing UTNs (preferably with a long lasting product like KO-Tab 1-2-3^®^[13]) for lower socio-economic groups, as well as educational programmes for the entire population, including better-situated families, to reinforce the knowledge about the danger of *P. falciparum *infection.

## Authors' contributions

JNG designed the study, collected data, analysed data and prepared the first draft of the manuscript. Some of the data presented here form part of JNG's doctoral thesis. NGS contributed to the design of the study, the data analysis and the writing-up. MLD, SO, LBB, UDK, MP, BL and SI contributed to the study design, the acquisition of data, and the writing-up. PGK and MPG contributed to designing, interpreting and writing-up of the study. All authors read and approved the final manuscript.
